# Moderate SCRIB Expression Levels Correlate with Worse Prognosis in OPSCC Patients Regardless of HPV Status

**DOI:** 10.3390/cells13121002

**Published:** 2024-06-08

**Authors:** Lucija Lulić, Ivana Šimić, Ksenija Božinović, Ena Pešut, Luka Manojlović, Magdalena Grce, Emil Dediol, Ivan Sabol, Vjekoslav Tomaić

**Affiliations:** 1 Division of Molecular Medicine, Ruđer Bošković Institute, Bijenička Cesta 54, 10000 Zagreb, Croatia; 2 Division of Molecular Biology, Ruđer Bošković Institute, Bijenička Cesta 54, 10000 Zagreb, Croatia; 3 Department of Pathology and Cytology, University Hospital Dubrava, 10000 Zagreb, Croatia; 4 Department of Maxillofacial Surgery, University Hospital Dubrava, 10000 Zagreb, Croatia

**Keywords:** HPV, survival, SCRIB, NHERF2, DLG1, HNSCC, E6

## Abstract

Head and neck cancers rank as the sixth most prevalent cancers globally. In addition to traditional risk factors such as smoking and alcohol use, human papillomavirus (HPV) infections are becoming a significant causative agent of head and neck cancers, particularly among Western populations. Although HPV offers a significant survival benefit, the search for better biomarkers is still ongoing. In the current study, our objective was to investigate whether the expression levels of three PDZ-domain-containing proteins (SCRIB, NHERF2, and DLG1), known HPV E6 cellular substrates, influence the survival of HNSCC patients treated by primary surgery (n = 48). Samples were derived from oropharyngeal and oral cancers, and HPV presence was confirmed by PCR and p16 staining. Clinical and follow-up information was obtained from the hospital database and the Croatian Cancer registry up to November 2023. Survival was evaluated using the Kaplan–Meier method and Cox proportional hazard regression. The results were corroborated through the reanalysis of a comparable subset of TCGA cancer patients (n = 391). In conclusion, of the three targets studied, only SCRIB levels were found to be an independent predictor of survival in the Cox regression analysis, along with tumor stage. Further studies in a more typical Western population setting are needed since smoking and alcohol consumption are still prominent in the Croatian population, while the strongest association between survival and SCRIB levels was seen in HPV-negative cases.

## 1. Introduction

Head and neck cancers (HNCs) are the sixth most common cancers worldwide, with an incidence of around 600 000 cases per year [1,2]. Head and neck squamous cell carcinomas (HNSCCs), derived from the mucosal epithelium in the upper aerodigestive tract, represent more than 90% of HNCs [1]. These cancers share similar risk factors, including smoking, tobacco chewing, alcohol consumption (~75% of HNSCC cases), and human papillomavirus (HPV) (~25% of HNSCC cases) [3,4,5]. HPV is particularly linked to oropharyngeal squamous cell carcinoma (OPSCC), which frequently occurs in the tonsils, base of the tongue, soft palate, and uvula [6]. Up to 96% of these tumors are HPV16-positive (HPV16+) and, in contrast to cervical cancer, precancerous lesions are hardly detected [6]. HPV+ HNSCCs seem to differ from HPV-negative (HPV−) ones in genetic, epigenetic, and protein expression profiles, as well as epidemiological factors and clinical characteristics [7,8,9,10], but the currently available treatments for both groups are almost the same [7,11,12]. Similar to cervical infections, the majority of head and neck HPV infections (as high as 80%) are cleared by the immune system in healthy individuals within 6 to 20 months of the initial infection [3,13].

In general, a 5-year overall survival (OS) for HNCs is poor, typically ranging from 25% to 40% [7]. However, patients with HPV+ OPSCC have improved OS, regardless of the stage, up to approximately 80% at 5 years [7,14]. This difference is further emphasized in the 8th edition of the AJCC cancer staging guidelines, where HPV+ OPSCC is staged differently [15]. The incidence of HPV+ OPSCC is projected to increase in the following decades until the benefits of prophylactic HPV vaccination start to have an impact [6].

HPV, a double-stranded circular DNA virus, greatly contributes to various cancer types [10,16,17,18,19]. Over 450 HPV genomes have been sequenced and categorized into five genera [20,21]. The extensively studied alpha-papillomavirus (αHPV) species primarily target the mucosal epithelia of the anogenital and oral regions and can be categorized as high-risk (HR) or low-risk (LR), depending on their tumorigenic potential [22,23,24]. HR HPVs are associated with nearly 100% of cervical cancer cases, of which, the most notable are HPV16 and HPV18, which alone are responsible for approximately 70% of those cases. On the other hand, oropharyngeal cancers are primarily caused by HPV16 and show low or sporadic association with HPV18 or other HR HPV types [9,19,25].

Tumor suppressors p53 and pRb are two of the most defined cellular targets of E6 and E7, respectively [6,26,27]. Both are targeted by the oncoproteins for proteasome-mediated degradation [28,29]. Apart from promoting the degradation of p53, multiple studies have demonstrated that E6 interacts with various cellular targets, including proteins containing PDZ (PSD95/DlgA/Zo-1) domains [29,30]. Such PDZ-domain targets are often tumor suppressors and are involved in maintaining cellular homeostasis, growth, polarity, and proliferation control [31,32]. A good example of PDZ-domain-containing proteins includes SCRIB, NHERF2, and DLG1, which have been shown to be targeted by oncogenic HPV E6 via their C-terminal PDZ binding motifs (PBMs) in various tissue culture model systems [33,34]. Furthermore, recent analyses have demonstrated that DLG1 is translocated from intercellular contacts to the cytoplasm in both HPV16+ and HPV− OPSCC, suggesting that this phenotype could contribute to malignant development in these cancer types. Interestingly, the complete loss of SCRIB expression was much more evident in HPV16+ OPSCC than in poorly differentiated HPV− OPSCC, pointing out its importance for HPV-driven oncogenesis [35]. In addition, the targeting of NHERF2 also appears to be important for HPV-mediated carcinogenesis in the head and neck areas. A marked downregulation in the percentage of immunoreactive NHERF2 cancer cells in HPV16+ OPSCC, in comparison to well and moderately differentiated HPV− OPSCC, was evident [36]. 

Despite two decades of HPV research in HNSCCs, strategies for its prevention and early diagnosis are still lacking. The immunohistochemical identification of p16 is most often used as a surrogate for HPV+ cancers, but a strict correlation with cancer progression has not been established [37,38]. Thus, the aim of this study was to integrate SCRIB, NHERF2, and DLG1 immunohistochemistry (IHC) staining, along with HPV DNA/p16 detection, in a well-defined cohort of HNSCCs to assess the impacts of changes in the expression levels and cellular localization of those E6 substrates on patient outcomes.

## 2. Materials and Methods

For this retrospective analysis, 65 tissue sections preserved in formalin and embedded in paraffin (FFPE) were collected from the Pathology Department of the Clinical Hospital Dubrava. The inclusion criteria for the material search were tissue from oral or oropharyngeal primary tumors irrespective of HPV status. In eight cases, the residual FFPE block did not contain enough material for all subsequent analyses, three blocks contained only biopsy material from the same patient for which surgically resected material was already available, and one block was a secondary tumor in the same patient, leading to the exclusion of those 23 blocks from the final analysis. Furthermore, five tissue blocks belonged to patients who were treated by primary radiotherapy, from which only biopsy or post-radiation resection material was available. Those cases were excluded as well. In total, 48 blocks remained from patients treated with surgery at the Department of Maxillofacial Surgery in Clinical Hospital Dubrava, Zagreb, Croatia between 2011 and 2020. Informed consent was obtained from all patients at the time of treatment, and this study (Croatian Science Foundation Projects No. 4758 and 2246) was approved by the Bioethics Committees of the Clinical Hospital Dubrava and Ruđer Bošković Institute (EP-KBD-10.06.2014, BEP-55 48/2-2016, BEP-3748, and BEP-2-2014). The relevant information was extracted from medical records, including demographic (gender, age, and smoking and drinking status), clinical (tumor location, and treatment), and pathohistological (tumor grade, TNM, stage, and presence of angio/perineural invasion) records and follow-up (locoregional recurrence, distant metastases, emergence of secondary or previous primary tumors, and survival) information. Their medical records were reviewed, and information was updated until October 2023. Additional survival information was obtained from the Croatian Cancer Registry up to November 2023 for patients who were otherwise lost to follow-up. Tumor staging was conducted according to the guidelines outlined in the AJCC staging manual (8th edition) [15]. Alcohol and tobacco consumption as risk factors were reviewed; however, detailed alcohol consumption or smoking data were not available. 

Laboratory HPV DNA detection results were previously available [35,39]. Briefly, DNA was isolated from a separate tumor tissue sample obtained at the time of surgical treatment or FFPE block derived from the same primary tumor depending on availability (16/48 patients had fresh tissue available, in addition to FFPE tissue). Approximately 20 mg of fresh tissue or five to seven FFPE slices (10 µm) were used for DNA isolation. The DNA from fresh tissues was previously isolated using an EZ1 DNA Tissue Kit (Qiagen, Hilden, Germany) or GenElute-E Single Spin Tissue DNA Kit (Sigma-Aldrich). The DNA from FFPE tissues was isolated with a NucleoSpin^®^ DNA FFPE XS (Macherey–Nagel). The total DNA concentration of samples was assessed using a NanoPhotometer^®^ C40 (Implen, Munich, Germany).

The validation of DNA quality and the efficacy of the PCR were performed using the beta-globin (~150 bp) and beta-actin (~100 bp) housekeeping genes for fresh or FFPE tissues, respectively. PCR HPV detection was performed using PGMY, GP5+/GP6+, and LC primers, as described previously for the fresh material collected during surgery [39], or HPV consensus primers GP5+/GP6+ and SPF10 for the FFPE-derived DNA [35]. 

HPV involvement was indirectly detected using a CINtec^®^ p16 Histology kit system (Roche Holding AG, Basel, Switzerland) according to the manufacturer’s instructions, as described previously [35]. The combined results of HPV DNA and p16 positivity were used to separate HPV+ and HPV− tumors.

The tissue levels of DLG1, SCRIB, and NHERF2 proteins were also previously assessed [35,36]. Briefly, 10 µm of FFPE tissue sections were mounted on pretreated glass slides, deparaffinized in a xylene substitute (BioClear, Biognost, Zagreb, Croatia), and rehydrated using a graded ethanol series (100%, 95%, and 70%). Staining was performed using Santa Cruz Biotechnology (Dallas, TX, USA) mouse monoclonal antibodies DLG1-SAP 97 (2D11), SCRIB (C-6) at 1:20 dilution, and anti-NHERF2 antibody (C-2) at 1:30 dilution. Biotinylated secondary antibodies were included in a commercial kit EnVision^®^ + Dual Link System-HRP (Agilent Technologies, Santa Clara, CA, USA). Negative controls for each sample were processed in parallel without adding the primary antibody. The intensity of immunostaining was scored and graded by two independent pathologists: 0 (no staining), 1+ (low intensity), 2+ (medium intensity), and 3+ (strong intensity) for all proteins of interest.

MedCalc software (version 20.111) and R (version 4.2.2) were used for statistical analyses. Heatmap clustering of IHC results was performed in R using the pheatmap package (v1.0.12). Variables with limited case numbers were combined, as were distinct sub-levels of advanced-stage cancer (IVa, n = 25 and IVb, n = 10), to allow more meaningful comparisons. The survival outcomes, including overall survival (OS), encompassing patients deceased from any cause, disease-specific survival (DSS), involving only patients who died from the primary tumor treated at enrollment, and disease-free survival (DFS), indicating any recurrence, metastasis, or death from any cause, were recorded. The follow-up time in months was obtained from the date of surgery to last registered follow-up or death (or 1 November 2023 for the patients recorded as alive in the cancer registry) in the case of OS or DSS. The follow-up time in months from enrollment to disease recurrence, metastasis, or death from any cause was considered for DSS assessment. The survival differences were evaluated using the Kaplan–Meier method or Cox proportional hazard regression. Both univariate and multivariate models, containing variables significant in univariate analysis, were constructed. All *p*-values below 0.05 were considered statistically significant. 

The cancer genome atlas data (TCGA) [40] were assessed to corroborate the findings using the USCS Xena browser. A comparable subset of HNC cases from the TCGA cohort was extracted (n = 391), focusing on the comparable tumor anatomic subsites (oral cavity and oropharynx). Illumina mRNA sequencing expression levels of SCRIB, NHERF2 (i.e., SLC9A3R2), and DLG1 genes were examined, along with selected comparable patient information (gender, tumor grade, survival status, and p16 detection). R was used to construct similar heatmaps of mRNA expression, while the Xena browser was used to construct Kaplan–Meier survival curves for SCRIB expression for comparisons with the current dataset. Furthermore, the association of SCRIB with survival was separately assessed in HPV+ and HPV− subsets of TCGA data through THInCR [41].

## 3. Results

### 3.1. Patient and Tumor Characteristics

In total, 48 FFPE blocks of tumor tissues were obtained for IHC analyses and combined with complete medical records. Almost 90% of the study population were men (43/48, i.e., 89.6%). The patients were 57.3 years old on average (median: 37.5 and range: 31–84). Another prominent feature of the population was the high prevalence of smoking and drinking habits since only 31.3% were declared as never smokers/never drinkers (NSND: 15/48). Most tumors were detected in the oropharyngeal region (33/48, i.e., 68.8%). Among the anatomical sub-locations, the tonsil, base of the tongue, and oral tongue were predominant (Table 1). According to the 8th edition of the AJCC guidelines, most patients were treated for stage IV cancer (28/48, i.e., 58.3%) (Table 1). No distant metastases were observed at the baseline (M = 0). Local lymph node involvement was found in the majority of patients with neck dissection pathology results (pN > 0 and 33/42, i.e., 78.6%). Histologically, the majority of patients exhibited grade II tumors (18/48, i.e., 37.5%).

The samples were divided into two groups based on HPV DNA detection and p16 status: 12 (25%) samples with HPV+ (associated) and 36 (75%) samples with HPV− HNSCCs. The HPV+ patients were marginally younger than the HPV− group (55.3 ± 11.1 vs. 57.9 ± 9.4 years). The HPV+ patients had lower overall cancer stage than HPV− patients (8th edition AJCC guidelines). Interestingly, all p16-positive cases were also HPV DNA-positive, even for oral cancer cases (n = 2), while there were additional DNA-positive cases without p16 positivity. Almost half of the NSND patients were HPV-associated (7/15, i.e., 46.7%), while only 11.5% cases in the SD group were HPV-associated, which was marginally statistically different. 

### 3.2. Immunostaining of DLG1, SCRIB and NHERF2 Proteins

**Figure 1:**
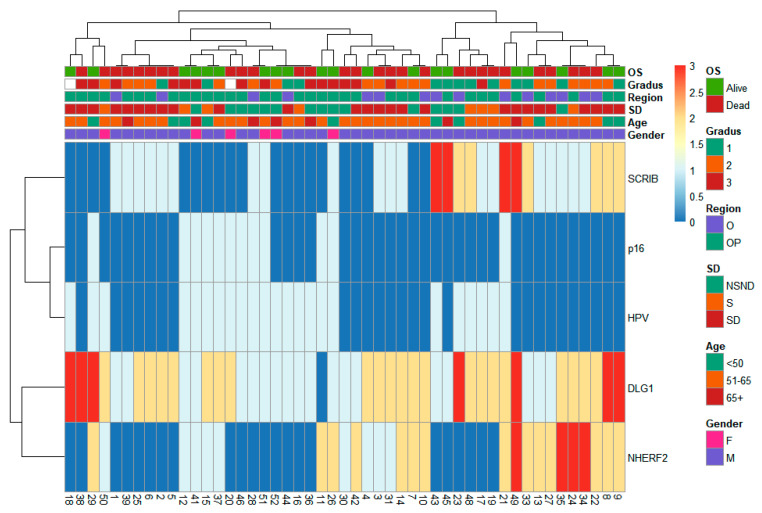
Summary of various experimental results and clinical parameters. IHC staining intensity for SCRIB, NHERF2, and DLG1 expression, together with p16 and HPV DNA positivity, does not separate cancer cases in noticeable patterns from other patient characteristics. OS: overall survival; O: oral; OP: oropharyngeal; NSND: never smoker and never drinker; S: smoker; and SD: smoker and drinker.

Representative images of different IHC staining patterns were individually reported previously [35,36]. Within this combined dataset, most tumors had some DLG1 staining, while SCRIB and NHERF2 protein expression levels were often completely absent (Table 2). Only SCRIB was statistically more often absent in HPV-associated tumors (66.7% vs. 30.6%, *p* = 0.028 based on a chi-squared test). The staining patterns were not noticeably different. No discernible patterns of IHC positivity of targeted proteins could be observed (Figure 1), which is in accordance with the data obtained from the TCGA HNSCC cohort (Supplementary Figure S1).

### 3.3. NHERF2 and SCRIB Tissue Expression Affect Patient Survival

**Figure 2:**
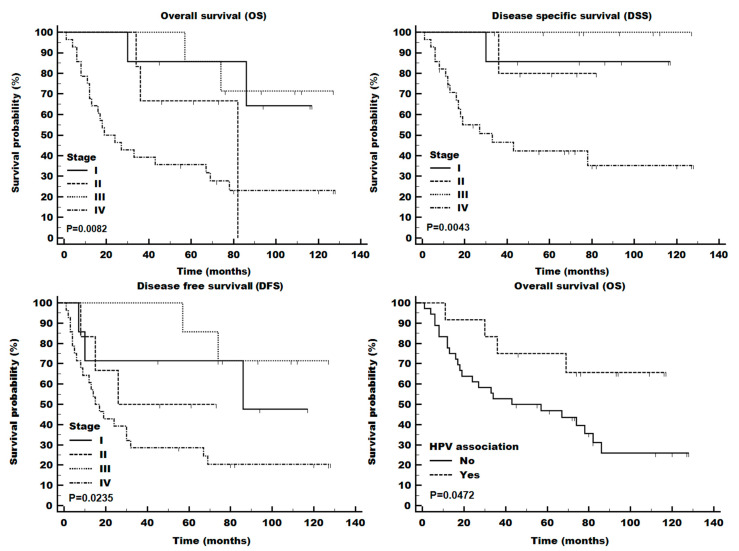
Tumor stage (8th ed AJCC guidelines) and HPV status affected patient survival. Tumors with p16 staining and HPV DNA were considered HPV-mediated. OS: overall survival; DSS: disease-specific survival; DFS: disease-free survival.

**Figure 3:**
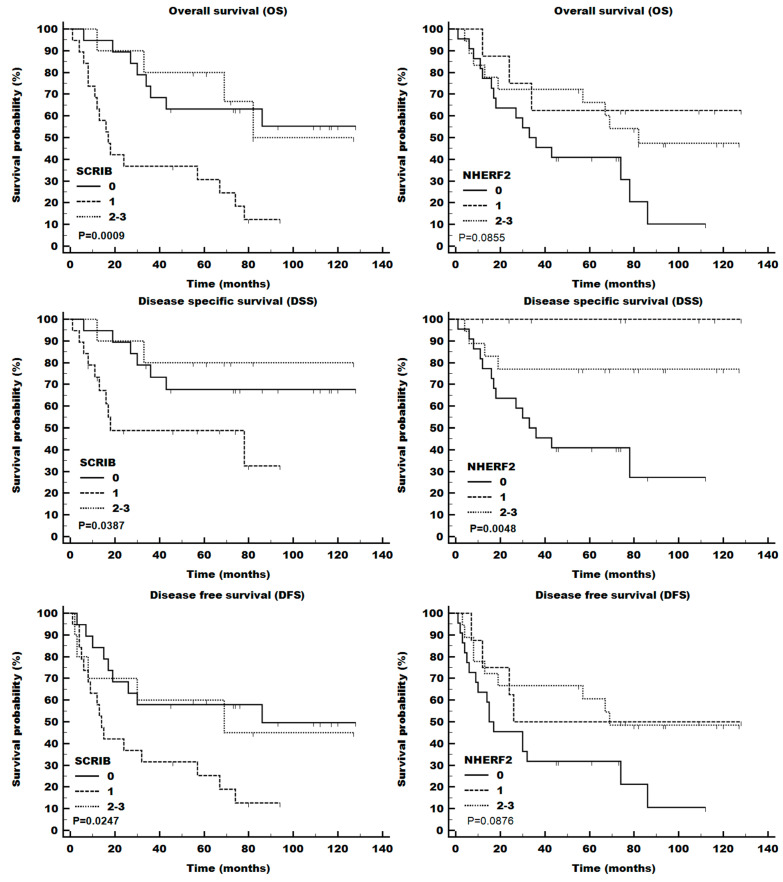
Overall (OS), disease-specific (DSS), and disease-free (DFS) survival of patients with different expressions of SCRIB and NHERF2 proteins detected by IHC.

During the follow-up observation period (median of 56 months and maximum of 128 months), 28 of 48 patients died, of which 10 died of unrelated causes (Table 1). The overall tumor stage was significantly associated with all measures of survival (OS, DSS, and DFS; Figure 2), while HPV-associated tumors exhibited only somewhat improved overall survival (*p* = 0.0408 (KM test); Figure 2, bottom right panel). Similarly, the intensity of SCRIB staining also demonstrated significant differences in OS (Figure 3), while NHERF2 depletion was only partially associated with decreased survival. DLG1 intensity was not significantly associated with a change in survival (Supplementary Figure S2).

**Figure 4:**
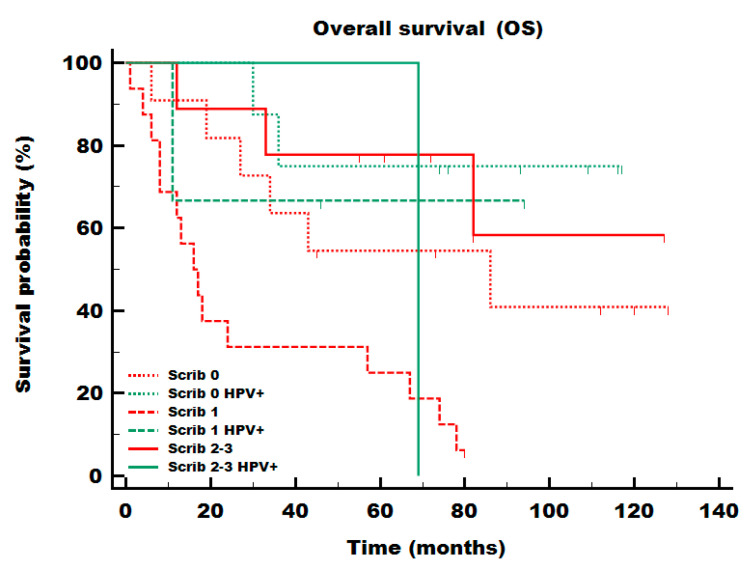
Combination of HPV and SCRIB expression levels in HNSCCs. Intermediate SCRIB levels (dashed line) were more deleterious in both HPV-associated (green lines) and p16-negative (red lines) tumors when considering the overall survival.

Cox proportional hazard regression analysis was conducted for relevant parameters and revealed that stage and SCRIB levels were independent prognostic factors of OS in multivariate analysis (Table 3). Interestingly, SCRIB staining of low intensity was more hazardous (HR = 2.66, 95% CI 1.06–6.64) than either lower (0: referent HR = 1) or higher levels (2–3: HR = 0.55, 95% CI 0.16–1.88). However, the same unusual pattern where intermediate levels are more hazardous could be seen in the Kaplan–Meier survival analysis of the TCGA HNSCC cohort (Supplementary Figure S3). SCRIB levels correlated with survival both in HPV-associated tumors as well as in HPV− tumors (Figure 4). The THInCR resource was used to assess how SCRIB expression levels are associated with OS within the HPV-associated and HPV− tumor subsets of the TCGA HNSCC cohort (Supplementary Figure S4).

## 4. Discussion

HNSCCs rank among the top six most common cancers globally, known for their aggressive nature characterized by advanced local disease and limited response to treatment options [1]. Currently, reliable biomarkers that could predict the development of the disease at early stages are still lacking, and the search for such predictive markers continues [42]. 

The male population is often found to be more prone to both HPV+ and HPV− HNSCCs [43], and this was also confirmed by our study. Possible reasons for such distribution could be hormone-related or might be affected by different susceptibilities to infection between genders [43]. In addition, men have a higher tendency to report increased numbers of sexual partners [6]. Furthermore, tobacco and alcohol consumption as high-risk factors are culturally and traditionally more male-associated, which could explain the discrepancy. 

Most cases of HPV+ OPSCC remain in those <65 years of age [6], and our study coincides with this finding. At the same time, this group is also the most common age group, making population-scale conclusions challenging.

Of the three PDZ-domain-containing proteins studied, only SCRIB exhibited a statistically significant association with patient survival. The association remained significant in multivariate Cox regression analysis, where both tumor stages and SCRIB were found to be independent predictors of survival (Table 3). While we have previously shown that HPV E6 leads to the degradation of SCRIB protein levels [35], apparently, tumors with low SCRIB levels (especially without HPV, Figure 4) have worse prognosis than either extreme. Importantly, one needs to bear in mind that the loss of cell polarity is a hallmark of cancer, which does not need to be necessarily linked to HPV presence. Therefore, it is expected that the fluctuations of these PDZ-domain-containing protein expression levels occur in both HPV+ and HPV− tumors [44]. While this finding might be due to some chance effect in a small population, it was very interesting to note that in the TCGA cohort, the middle-expression group had a worse prognosis than the top- or bottom-expression groups, with the groups starting to diverge noticeably after 30 months of follow-up (Supplementary Figure S2). Despite being noticeable, the difference was not statistically significant, which might be due to the stronger survival benefits of HPV in the US population. The information about SCRIB is further confounded by its diverse roles, where it is downregulated in some cancers and upregulated in others [45].

Tobacco and alcohol are well-known risk factors in HNSCCs, along with HPV, especially in OPSCC, which are becoming more prominent [46]. Although in this study, most tumors were located in the oropharyngeal region (68.8%), which is a predilection site for HPV-associated cancers [47], the survival benefit of HPV+ tumors was marginal (OR: 0.36, *p* = 0.057). Apparently, extensive tobacco and alcohol use overshadowed or at least masked the positive effects of HPV in this population. Our previous studies on Croatian HNSCC patients from a second unrelated hospital spanning a similar period also found smoking and alcohol consumption to be of greater importance than HPV presence [48]. Furthermore, a large cohort study reported different risk profiles for HNC survival based on a combination of HPV status and smoking history [49]. This supports the observation that the detrimental effects of tobacco and alcohol use may overshadow or mask the potential protective effects of HPV in certain populations. Those observations would likely also hold true for other populations where alcohol and smoking use still did not decline to the levels of more typical Western populations, where HPV is starting to predominate as a risk factor. In addition, the effect of HPV on OS in non-OPSCC is controversial [7]. According to several studies, there was no prognostic benefit of HPV in non-OPSCC, but a few studies [50] found HPV positivity as a good prognostic factor. Despite the introduction of HPV vaccination and proven efficacy against oral HPV infections, HPV+ OPSCC rates are still likely to rise, while smoking cessation campaigns will hopefully lead to a further decline in non-HPV-mediated HNSCCs over the next 20–30 years.

The limitations of our study should be noted. A relatively small number of patients with available FFPE material for all the analyses could affect the results. The identification of HPV presence by a combined assessment of p16 and HPV DNA detection is a strength since either of the methods is often suboptimal when used alone. This was recently confirmed by the fact that survival benefits could only be seen in p16 and DNA double-positive cases [38]. 

In conclusion, we have shown that the PDZ-domain-containing protein SCRIB might be a worthwhile target for further investigation in the context of HNSCCs that acted as an independent prognostic factor. The difference between HPV-associated and HPV-negative HNSCCs should be further investigated in populations where HPV is becoming a predominant risk factor. 

## Figures and Tables

**Table 1:** Patient characteristics.

Variable	Factor	Total (n = 48)	HPV+ ^1^ (n = 12)	HPV− ^1^ (n = 36)	*p*-Value
		N (%)	N (%)	N (%)	
Gender	M	43 (89.6)	8 (66.7)	35 (97.2)	0.0109 ^2^
	F	5 (10.4)	4 (33.3)	1 (2.8)	
Age	Mean (SD)	57.3 (9.8)	55.3 (11.1)	57.9 (9.4)	0.4281 ^3^
	Median (IQR)	57.5 (52.0–62.0)	59 (48.0–60.5)	57 (52.5–62.0)	
	Min–max	31–84	32–73	31–84	
	0–64	40 (83.3)	10 (83.3)	30 (83.3)	
	65+	8 (16.7)	2 (16.7)	6 (16.7)	
Lifestyle ^5^	NSND	15 (31.3)	7 (58.3)	8 (22.2)	0.0425 ^4^
	S	7 (14.6)	2 (16.7)	5 (13.9)	
	SD	26 (54.2)	3 (25)	23 (63.9)	
Site	Oral	15 (31.3)	2 (16.7)	13 (36.1)	0.2130 ^4^
	Gingiva	4 (8.3)	1 (8.3)	3 (8.3)	
	Tongue	6 (12.5)	0 (0)	6 (16.7)	
	Retromolar	3 (6.3)	0 (0)	3 (8.3)	
	Sublingual	2 (4.2)	1 (8.3)	1 (2.8)	
	Oropharyngeal	33 (68.8)	10 (83.3)	23 (63.9)	
	Base of tongue	13 (27.1)	0 (0)	13 (36.1)	
	Soft palate	1 (2.1)	0 (0)	1 (2.8)	
	Posterior pharyngeal wall	3 (6.3)	0 (0)	3 (8.3)	
	Tonsil	16 (33.3)	10 (83.3)	6 (16.7)	
Grade	1	13 (27.1)	1 (8.3)	12 (33.3)	0.0668 ^4^
	2	18 (37.5)	3 (25)	15 (41.7)	
	3	15 (31.3)	7 (58.3)	8 (22.2)	
	Missing data	2 (4.2)	1 (8.3)	1 (2.8)	
Stage	I	7 (14.6)	5 (41.7)	2 (5.6)	0.0026 ^4^
	II	6 (12.5)	2 (16.7)	4 (11.1)	
	III	7 (14.6)	3 (25)	4 (11.1)	
	IV	28 (58.3)	2 (16.7)	26 (72.2)	
Invasion	No invasion	27 (56.3)	7 (58.3)	20 (55.6)	0.6345 ^4^
	Perineural	13 (27.1)	4 (33.3)	9 (25)	
	Angio + perineural	8 (16.7)	1 (8.3)	7 (19.4)	
HPV DNA	Negative	26 (54.2)	0 (0)	26 (72.2)	<0.0001 ^4^
	Positive	22 (45.8)	12 (100)	10 (27.8)	
Therapy	Surgery	15 (31.3)	1 (8.3)	14 (38.9)	0.0504 ^4^
	Surgery + C/RT	33 (68.8)	11 (91.7)	22 (61.1)	
Follow-up status	Disease-free	20 (41.7)	8 (66.7)	12 (33.3)	0.1184 ^4^
	Dead from other causes	10 (20.8)	1 (8.3)	9 (25)	
	Dead from disease	18 (37.5)	3 (25)	15 (41.7)	
Follow-up time (months)	Mean (SD)	56.2 (39.7)	72.6 (35.3)	50.8 (40)	0.0993 ^3^
	Median (IQR)	56 (17.5–82.0)	75 (41.0–101.5)	44 (14.5–79.0)	
	Min–max	1–128	11–117	1–128	
Follow-up events	Local recidive	10 (20.8)	2 (16.7)	8 (22.2)	0.6847 ^4^
	Distant metastasis	10 (20.8)	3 (25)	7 (19.4)	0.6847 ^4^
	Second primary tumor	13 (27.1)	0 (0)	13 (36.1)	0.0210 ^2^

^1^ HPV-positive cases were determined by combined HPV DNA and p16 positivity, and *p*-values were determined by the ^2^ Fischer’s exact test, ^3^ *t*-test, or ^4^ chi-squared test. ^5^ NSND: never smoker and never drinker; S: smoker; and SD: smoker and drinker.

**Table 2:** SCRIB, NHERF2, and DLG1 protein levels on IHC staining.

Variable	Factor	Total (n = 48)	HPV+ (n = 12)	HPV− (n = 36)	Chi-Squared *p*-Value
		N (%)	N (%)	N (%)	
SCRIB intensity	0	19 (39.6)	8 (66.7)	11 (30.6)	0.0284
	1	19 (39.6)	3 (25)	16 (44.4)	
	Strong (2–3)	10 (20.8)	1 (8.3)	9 (25)	
	2	6 (12.5)	0 (0)	6 (16.7)	
	3	4 (8.3)	1 (8.3)	3 (8.3)	
SCRIB pattern	Loss of expression	19 (39.6)	8 (66.7)	11 (30.6)	0.0586
	Cytoplasm only	23 (47.9)	4 (33.3)	19 (52.8)	
	Cytoplasm and membrane	6 (12.5)	0 (0)	6 (16.7)	
NHERF2 intensity	0	22 (45.8)	4 (33.3)	18 (50)	0.1934
	1	8 (16.7)	4 (33.3)	4 (11.1)	
	Strong (2–3)	18 (37.5)	4 (33.3)	14 (38.9)	
	2	14 (29.2)	4 (33.3)	10 (27.8)	
	3	4 (8.3)	0 (0)	4 (11.1)	
NHERF2 pattern	Loss of expression	22 (45.8)	4 (33.3)	18 (50)	0.3207
	Cytoplasm only	26 (54.2)	8 (66.7)	18 (50)	
DLG1 intensity	Weak (0–1)	20 (41.7)	7 (58.3)	13 (36.1)	0.1809
	0	1 (2.1)	1 (8.3)	0 (0)	
	1	19 (39.6)	6 (50)	13 (36.1)	
	Strong (2–3)	28 (58.3)	5 (41.7)	23 (63.9)	
	2	21 (43.8)	4 (33.3)	17 (47.2)	
	3	7 (14.6)	1 (8.3)	6 (16.7)	
DLG1 pattern	Loss of expression	1 (2.1)	1 (8.3)	0 (0)	0.0814
	Cytoplasm only	35 (72.9)	10 (83.3)	25 (69.4)	
	Cytoplasm and membrane	12 (25)	1 (8.3)	11 (30.6)	

**Table 3:** Uni- and multivariate Cox proportional hazard regression analysis of parameters influencing overall survival.

Variable ^1^	Factor	Total	Univariate			Multivariate		
		N (%)	HR	CI	P ^1^	HR	CI	P ^1^
Gender	M	43 (89.6)	1					
	F	5 (10.4)	0.26	0.04–1.96	0.1937			
Age	0–64	40 (83.3)	1					
	65+	8 (16.7)	1.01	0.35–2.97	0.9843			
Lifestyle ^2^	NSND	15 (31.3)	1					
	S	7 (14.6)	1.59	0.48–5.24	0.4448			
	SD	26 (54.2)	1.95	0.77–4.95	0.162			
Site ^3^	O	15 (31.3)	1					
	OP	33 (68.8)	0.67	0.31–1.45	0.3052			
Grade	1	13 (27.1)	1					
	2	18 (37.5)	2.47	0.88–6.92	0.0865			
	3	15 (31.3)	1.91	0.64–5.72	0.2475			
	Unknown	2 (4.2)	1.16	0.14–9.94	0.8927			
**Stage**	I	7 (14.6)	1			1		
	II	6 (12.5)	2.36	0.39–14.51	0.3453	3.14	0.5–19.88	0.2247
	III	7 (14.6)	0.94	0.13–6.65	0.9474	0.96	0.13–6.87	0.9656
	IV	28 (58.3)	5.12	1.19–22.05	**0.0285**	5.21	1.15–23.68	**0.0327**
HPV ^4^	Negative	36 (75)						
	Positive	12 (25)	0.36	0.12–1.04	0.0578			
DLG1	0–1	20 (41.7)	1					
	2–3	28 (58.3)	1.29	0.6–2.81	0.5158			
**SCRIB**	0	19 (39.6)	1			1		
	1	19 (39.6)	3.88	1.63–9.25	**0.0022**	2.66	1.06–6.64	**0.0369**
	2–3	10 (20.8)	0.98	0.29–3.25	0.9691	0.55	0.16–1.88	0.3371
NHERF2	0	22 (45.8)	1					
	1	8 (16.7)	0.35	0.1–1.19	0.0929			
	2–3	18 (37.5)	0.48	0.21–1.11	0.0845			

^1^ Variables exhibiting significant differences are highlighted in bold. ^2^ NSND: never smoker and never drinker; S: smoker; SD: smoker and drinker; ^3^ O: oral region; and OP: oropharyngeal region. ^4^ HPV+ cases were determined by combined HPV DNA and p16 positivity.

## Data Availability

The original contributions presented in this study are included in the article/Supplementary Materials; further inquiries can be directed to the corresponding author/s. Publicly available TCGA data are available through the University of Santa Cruz’s Xena browser https://xena.ucsc.edu/ (accessed on 12 March 2024).

## Supplementary Materials

The following supporting information can be downloaded at: https://www.mdpi.com/article/10.3390/cells13121002/s1, Figure S1. Illumina sequencing-derived mRNA expression levels of SCRIB, NHERF2, and DLG1 genes.; Figure S2. Overall (OS), disease-specific (DSS), and disease-free (DFS) survival of patients with different expressions of DLG1; Figure S3. Survival analysis of 3 levels of SCRIB mRNA expression in the TCGA HNSCC cohort, focusing on oral and oropharyngeal cancer cases (n = 391). Figure S4. Survival analysis of 3 levels of SCRIB mRNA expression in the TCGA HNSCC cohort segregated by HPV positivity created by THInCR.
