# Supplementary material for: Moderate SCRIB Expression Levels Correlate with Worse Prognosis in OPSCC Patients Regardless of HPV Status
Source: Cells. 2024 Jun 8;13(12):1002. doi: 10.3390/cells13121002 (PMC11201649; doi:10.3390/cells13121002)
Supplement: Supplementary file 1 [file cells-13-01002-s001.zip › cells-2986077-supplementary.pdf]

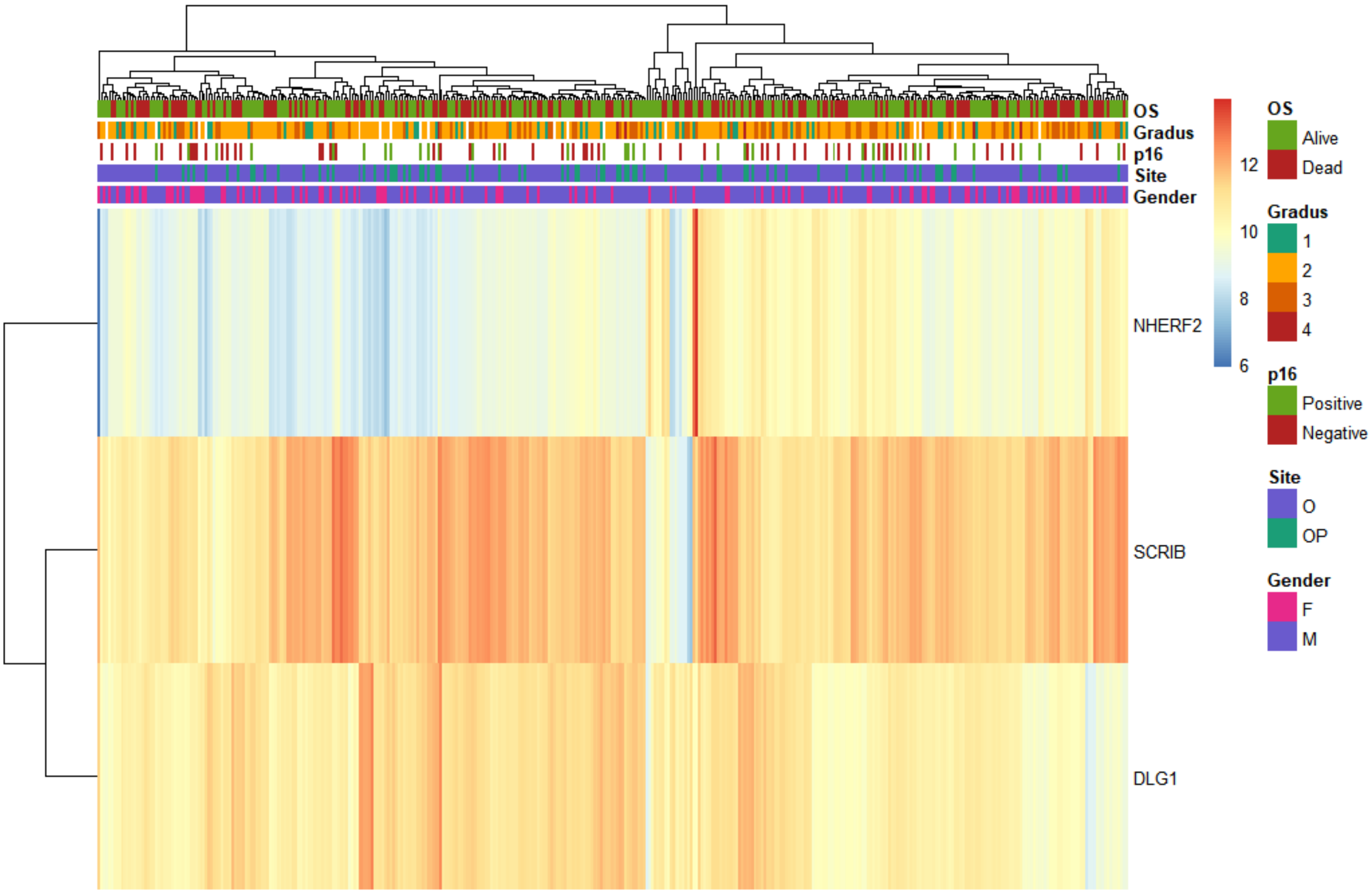

Supplementary Figure S1. Illumina sequencing derived mRNA expression levels of SCRIB, NHERF2 and DLG1, genes. Since most cases did not contain HPV information it was not possible to include it in clustering, but it is shown as column annotation. Each column is a patient from the TCGA HNSCC cohort focusing on oral and oropharyngeal cancer cases (n=391).

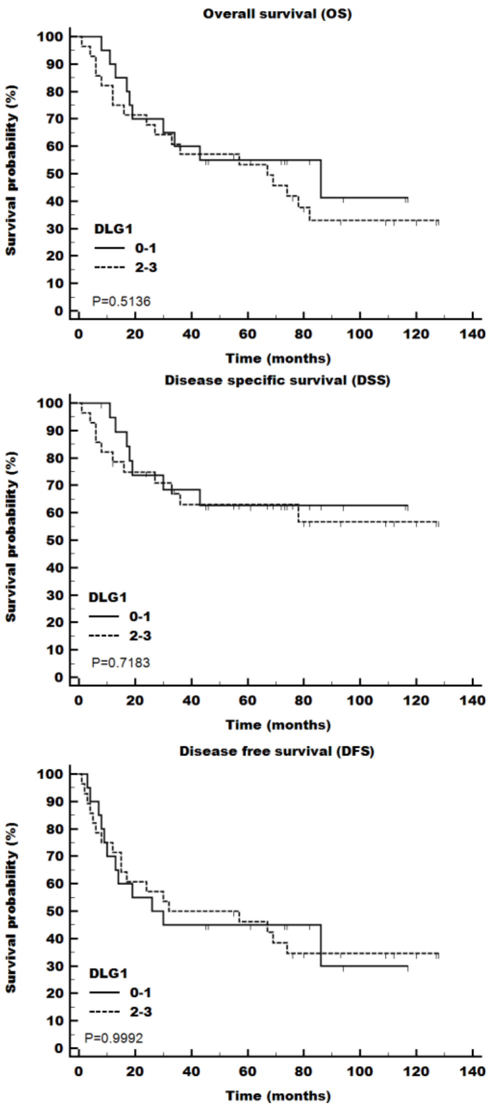

Supplementary Figure S2. Overall (OS), disease specific (DSS) and disease-free survival (DFS) of patients with different expression of DLG1.

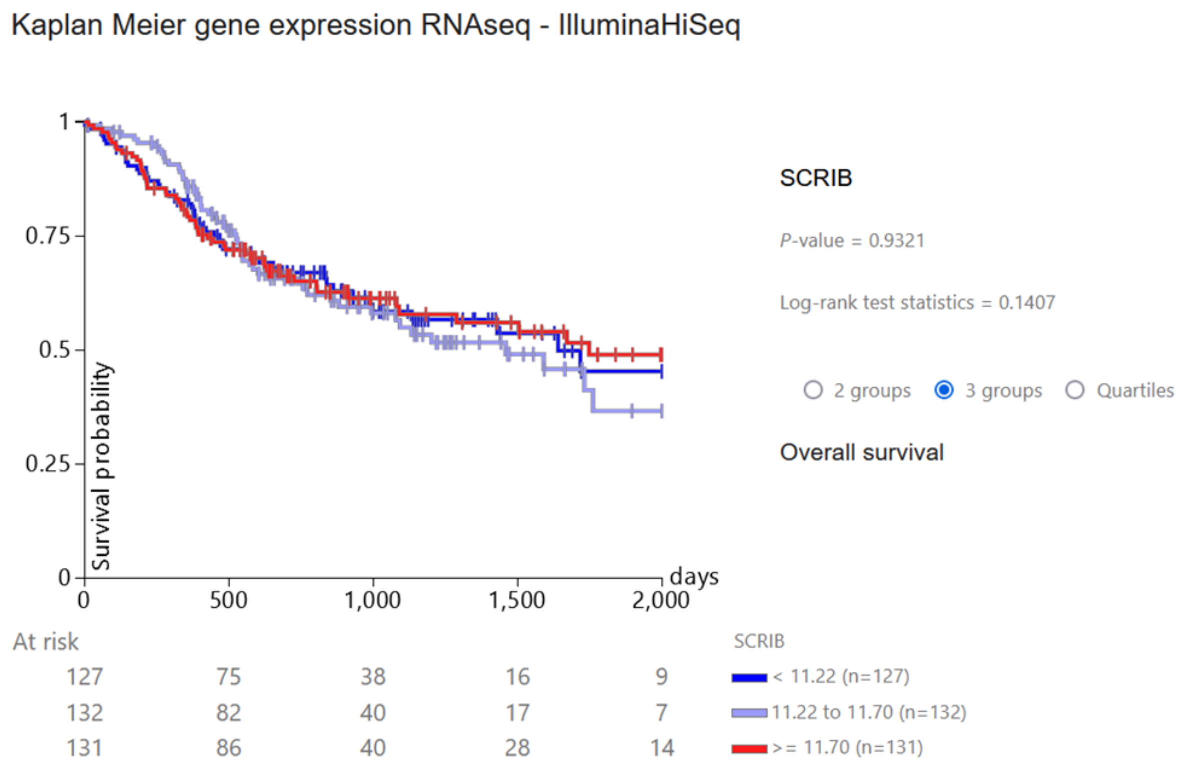

Supplementary Figure S3. Survival analysis of 3 levels of SCRIB mRNA expression in the TCGA HNSCCs cohort focusing on oral and oropharyngeal cancer cases (n=391). Intermediate levels of SCRIB expression show somewhat worse prognosis than either higher or lower values, but the difference was not statistically significant.

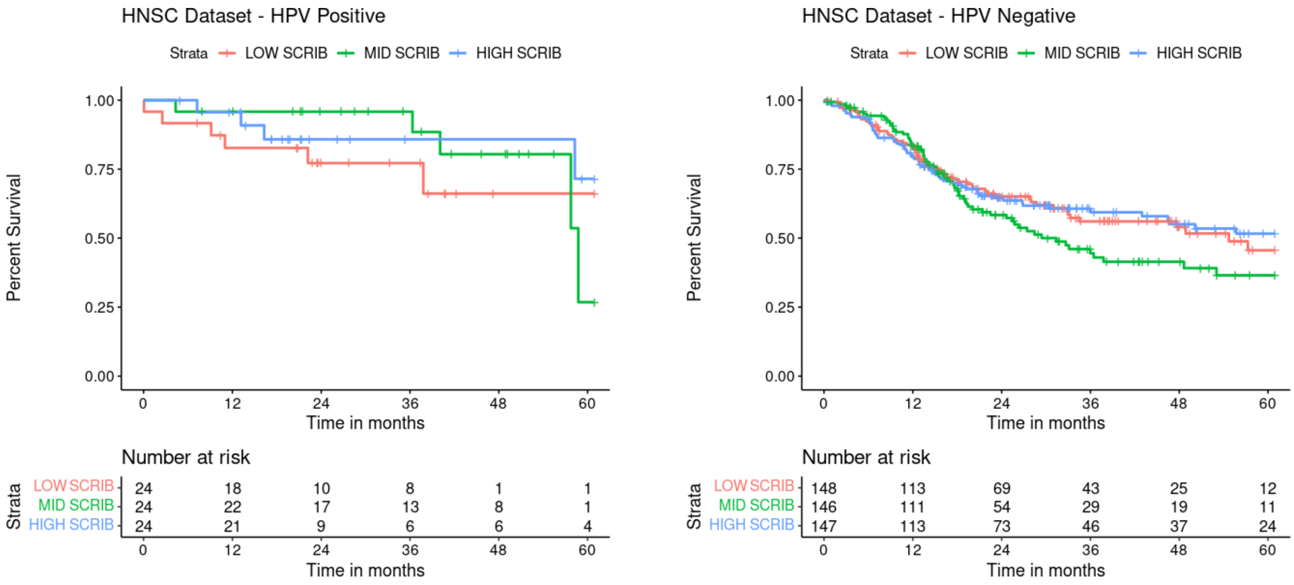

Supplementary Figure S4. Survival analysis of 3 levels of SCRIB mRNA expression in the TCGA HNSCCs cohort segregated by HPV positivity created by THInCR. In the HPV positive subset (left), intermediate levels of SCRIB expression had the worst overall survival at 60 months, but not at earlier timepoint. As before intermediate SCRIB levels in HPV− subset (right) somewhat more clearly indicated worse prognosis than either higher or lower values. The differences did not reach statistical significance in either set.
